# Exceptional Diversity, Maintenance of Polymorphism, and Recent Directional Selection on the *APL1* Malaria Resistance Genes of *Anopheles gambiae*

**DOI:** 10.1371/journal.pbio.1000600

**Published:** 2011-03-08

**Authors:** Susan M. Rottschaefer, Michelle M. Riehle, Boubacar Coulibaly, Madjou Sacko, Oumou Niaré, Isabelle Morlais, Sekou F. Traoré, Kenneth D. Vernick, Brian P. Lazzaro

**Affiliations:** 1 Department of Entomology, Cornell University, Ithaca, New York, United States of America; 2 Department of Microbiology, University of Minnesota, Saint Paul, Minnesota, United States of America; 3 Malaria Research and Training Center, University of Bamako, Bamako, Mali; 4 Laboratoire de Recherche sur le Paludisme, Institut de Recherche pour le Développement IRD-OCEAC, Yaoundé, Cameroun; 5 Unit of Insect Vector Genetics and Genomics, Institut Pasteur, Paris, France; Stanford University, United States of America

## Abstract

The three-gene *APL1* locus encodes essential components of the mosquito immune defense against malaria parasites. *APL1* was originally identified because it lies within a mapped QTL conferring the vector mosquito *Anopheles gambiae* natural resistance to the human malaria parasite, *Plasmodium falciparum*, and *APL1* genes have subsequently been shown to be involved in defense against several species of *Plasmodium*. Here, we examine molecular population genetic variation at the *APL1* gene cluster in spatially and temporally diverse West African collections of *A. gambiae*. The locus is extremely polymorphic, showing evidence of adaptive evolutionary maintenance of genetic variation. We hypothesize that this variability aids in defense against genetically diverse pathogens, including *Plasmodium*. Variation at *APL1* is highly structured across geographic and temporal subpopulations. In particular, diversity is exceptionally high during the rainy season, when malaria transmission rates are at their peak. Much less allelic diversity is observed during the dry season when mosquito population sizes and malaria transmission rates are low. *APL1* diversity is weakly stratified by the polymorphic 2La chromosomal inversion but is very strongly subdivided between the M and S “molecular forms.” We find evidence that a recent selective sweep has occurred at the *APL1* locus in M form mosquitoes only. The independently reported observation of a similar M-form restricted sweep at the *Tep1* locus, whose product physically interacts with APL1C, suggests that epistatic selection may act on these two loci causing them to sweep coordinately.

## Author Summary

Immune defense genes are sometimes highly variable in host populations, reflecting selective pressure to combat diverse pathogens. In other instances, where there are only a few dominant pathogens, natural selection may favor only one or a few defense alleles. Here, we show that both adaptive strategies can occur in the same genes under different circumstances. We examined diversity in the *APL1* genes of the human malaria vector mosquito *Anophleles gambiae*, which play a role in defense against malaria parasites. We found that the *APL1* genes are exceptionally polymorphic, being 10-fold more diverse than typical *A*. *gambiae* genes. The distribution of *APL1* allelic diversity, however, is strongly structured depending on whether the genes are carried by the M or S “molecular forms” of the vector, which are thought to constitute newly forming species. We show that despite the evolutionary maintenance of *APL1* diversity in the S form of *A. gambiae*, there is evidence of strong recent directional selection on *APL1* genes in the M form. Independent research has shown that *Tep1*, a gene which encodes a protein that physically interacts with the APL1C protein, also harbors high allelic diversity in the S form and shows evidence of recent directional selection in the M form, suggesting that the evolutionary trajectories of the *Tep1* and *APL1* defense loci may be correlated.

## Introduction

Approximately 250 million human malaria cases are reported annually, most of them occurring in sub-Saharan Africa [1]. The vast majority of these are caused by the malaria parasite *Plasmodium falciparum*, vectored by the mosquito *Anopheles gambiae* [2]. However, many wild *A*. *gambiae* are genetically resistant to *P*. *falciparum* establishment and development [3],[4], suggesting that genetic variation in *A*. *gambiae* resistance has the potential to influence the dynamics of disease transmission among humans. Identification of the genes that moderate variation in mosquito resistance, and in particular those that may closely co-evolve with malaria parasites, could reveal attractive targets for control intervention and disease management. Despite its potentially great importance, however, remarkably little is known about molecular polymorphism in genes required for mosquito defense against malaria.

The *APL1* gene cluster is a strong candidate locus for determination of natural resistance to *P*. *falciparum* in wild populations of *A*. *gambiae*. The *APL1* cluster lies within a quantitative trait locus (QTL) controlling *P*. *falciparum* establishment that has been independently and recurrently mapped in both west and east African wild mosquito populations [3]–[6]. The *APL1* array is composed of three genes arranged head-to-tail in a 15 kilobase block, which have been denoted *APL1A*, *APL1B*, and *APL1C* and assigned VectorBase identification numbers AGAP007036, AGAP007035, and AGAP007033 [4],[7]. RNAi knockdown of *APL1A* causes increased mosquito susceptibility to *P*. *falciparum* infection [8] and RNAi knockdown of *APL1C* increases mosquito susceptibility to *P*. *berghei* and *P*. *yoelii* [4],[7]–[8]. Simultaneous RNAi knockdown of the three *APL1* homologs in the *A*. *gambiae* sister species *A*. *quadriannulatus* renders a normally resistant strain susceptible to *P*. *berghei* infection [9]. Transcriptional expression of all three paralogs is induced when mosquitoes feed on *Plasmodium*-laden bloodmeals, although the precise patterns of expression vary across the three genes [4]. *APL1A* transcription is regulated by the Imd/Rel2-S defense pathway [8]. *APL1C*, which shows the strongest and most temporally stable induction following a *Plasmodium*-laden bloodmeal, is regulated by the Toll/Cactus/Rel1 defense signaling pathway [7]. APL1C has recently been shown to complex with the anti-malaria *Anopheles* resistance protein LRIM1 [10] to regulate the activation of and to stabilize the opsonin TEP1, leading to *P*. *berghei* tagging and killing [11],[12]. The “G3” laboratory colony of *A*. *gambiae* segregates for divergent alleles of natural origin at *APL1A*, *APL1B*, and *APL1C* (denoted with superscripts 1 and 2; ref. [7]). Mosquitoes in the G3 colony that are homozygous for the *APL1A* ^2^/*APL1B* ^2^/*APL1C* ^2^ linkage group show marked resistance to *P*. *berghei* infection [7], suggesting that natural variation at *APL1* might be important for resistance to malaria in the field.

To date, population genetic studies focused on genes involved or hypothesized to be involved in *A*. *gambiae* immune defense have found little evidence for co-adaptive host-pathogen evolutionary dynamics [13]–[21], although these studies have generally been underpowered due to limited examination of small genes or gene fragments and by the unfortunate phylogenetic structure of *Anopheles*, where taxa sister to *A*. *gambiae* are too closely related for comparative tests to enjoy much power but more distant relatives are so far diverged that substitution at synonymous sites approaches saturation [14]. Despite these limitations, the molecular evolution of *Tep1* and *LRIM1*, whose products physically interact at least with APL1C, have been examined in some detail. *Tep1* is highly polymorphic at the amino acid and nucleotide levels, possibly due to the formation of chimeric alleles through paralogous gene conversion [18]. Divergent alleles of the *Tep1* gene have been shown to confer relative resistance and susceptibility to infection by *P*. *berghei* and *P*. *falciparum* [22]–[24]. In contrast, the level of polymorphism at *LRIM1* is typical of *A*. *gambiae* genes [14],[16], although *LRIM1* shows weak evidence of adaptive directional evolution in the *A*. *gambiae* sister species *A*. *arabiensis*. It has thus remained ambiguous whether the TEP1-LRIM1-APL1C complex evolves under diversifying selection, purifying selection, directional adaptation, or some combination of these forces.

Major structural variants of *APL1* genes have been previously reported [7], but the full extent of allelic polymorphism at *APL1* in wild mosquitoes has never been determined. In the present study, we conduct extensive population genetic sampling of west African *A*. *gambiae*, evaluating allelic diversity at *APL1* over time and space. We sequenced the *APL1A*, *APL1B*, and *APL1C* genes of wild *A*. *gambiae* collected from three sites in western sub-Saharan Africa: Bancoumana, Mali; Toumani-Oulena, Mali; and Makouchetoum, Cameroon. Bancoumana is in a relatively arid savannah near the capital city, Bamako. Toumani-Oulena is in a more humid forested region, and Makouchetoum is in a humid agricultural region near Foumbot. Samples were drawn from all three locations during the rainy season, when most malaria transmission happens, and additionally during the dry season from the Bancoumana population. We discovered exceptionally high genetic diversity at all three genes, with the majority of this variation observed during the rainy season. We find *APL1* genetic variation to be structured geographically, mediated by M/S “molecular form” (reviewed in [25]) and to a lesser degree by karyotype of the chromosomal inversion 2La. The evolutionary trajectory of *APL1* genes is highly significantly deviant from that of other genes in the *A*. *gambiae* genome and is generally consistent with adaptive maintenance of polymorphism in S form mosquitoes. At the same time, a recent and strong selective sweep has reduced diversity at the *APL1* locus in M form mosquitoes.

## Results

### Structure of the *APL1* Genes and Encoded Proteins

**Figure 1:**
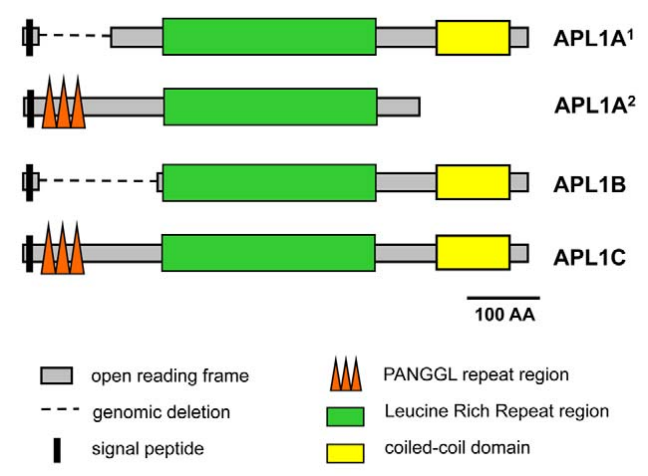
Schematic representation of proteins encoded by *APL1A*, *APL1B*, and *APL1C* genes. The two major structural variants of *APL1A* are shown separately. *APL1A^1^* alleles are characterized by the deletion of the PANGGL region. *APL1A^2^* alleles carry an early stop codon that eliminates the coiled-coil domain.

*APL1A*, *APL1B*, and *APL1C* are each composed of a small 5′ exon and longer second exon separated by a short intron [7]. Schematics of the encoded proteins are given in [7] and Figure 1. Each protein is characterized by an N-terminal signal peptide, a series of leucine-rich repeat (LRR) motifs spanning approximately 300 amino acids in the middle of the protein, and a coiled-coil domain at the C-terminus. *APL1A^2^* alleles encode a premature stop codon that terminates the protein downstream of the LRR domain, eliminating the C-terminal coiled-coil from the predicted mature protein. We observed 5 *APL1A* ^1^ alleles (out of 38 total sampled) in which the presumptive start codon has been mutated to ATA; it is unclear whether these alleles utilize an alternative ATG to initiate translation. *APL1C* alleles encode an N-terminal repeated motif of the amino acids P-A-N-G-G-L and related sequences (hereafter referred to as the PANGGL region). The *APL1B* gene does not have a PANGGL region. Interestingly, the PANGGL region is present in *APL1A* ^2^ alleles but absent from *APL1A* ^1^ [7]. In the course of the present study, we found that *APL1A* alleles of three species sister to *A*. *gambiae* (*A*. *arabiensis*, *A*. *quadriannulatus*, and *A*. *merus*) are all PANGGL-less and extremely similar to *APL1A* ^1^ alleles, suggesting that *APL1A* ^2^ alleles might be of recent evolutionary origin in *A*. *gambiae*. The deletion that eliminates PANGGL from *APL1B* relative to *APL1C* is 207 bp longer than and shares neither breakpoint with the insertion/deletion that distinguishes *APL1A* ^1^ from *APL1A* ^2^. Thus, there must have been at least two independent mutations resulting in either the gain or loss of the PANGGL region in *APL1* genes. The similarity in sequence between the PANGGL repeats and flanking regions of *APL1C* and *APL1A^2^* alleles, along with the apparent absence of *APL1A^2^* alleles in *A. merus*, *A*. *arabiensis*, and *A*. *quadriannulatus* (Figure S1), suggests that PANGGL repeats may have been introduced into the *APL1A* gene via paralogous conversion with *APL1C* in *A*. *gambiae*. Elevated mutation rate due to the repetitive structure and potentially ongoing exchange between *APL1C* and *APL1A^2^* might then generate allelic diversity in both genes. Paralogous gene conversion has similarly been hypothesized to explain the origin of divergent alleles of the *Tep1* gene in *A. gambiae* [18]. No function has been determined for the PANGGL repeat region, but convergence of a PANGGL-less structure in *APL1B* and *APL1A^1^* alleles and presence of PANGGL in *APL1C* and *APL1A^2^* alleles suggests that presence/absence of the PANGGL domain may alter *APL1* function and adaptive value. Testing this hypothesis will require manipulative experimentation.

### *APL1* Genes Are Exceptionally Polymorphic

Species-level nonsynonymous (amino acid altering) polymorphism is extraordinarily high in *APL1A*, *APL1B*, and *APL1C*, with per-nucleotide estimates of nonsynonymous diversity (π_non_) of 5.9%, 3.1%, and 2.4%, respectively (Table 1). These values are approximately 10-fold higher than what is typically observed in *A*. *gambiae* genes, including those with immune function ([14]–[17],[ 19]–[21], but see [18]). There is some sharing of polymorphism across the *APL1* genes, consistent with paralogous gene conversion that may elevate diversity within genes by introducing blocks of sequence from neighboring loci. Potential conversion events are unsurprisingly most abundant in the LRR region. The majority of the observed polymorphism across the *APL1* genes, however, cannot be explained by origin through recent conversion.

**Table 1:** Population genetic parameter estimates at the *APL1* locus in four collections.

Collection	n^a^	bp^b^	π_tot_ c	θ_tot_ d	TajD^e^	π_syn_ f	π_non_ g
***APL1A***							
Bancoumana dry	19	1,669	0.019	0.032	−1.734	0.033	0.016
Bancoumana rainy	9	1,665	0.048	0.054	−0.587	0.086	0.039
Toumani-Oulena	12	1,541	0.084	0.074	0.668	0.119	0.066
Makouchetoum	8	1,541	0.088	0.078	0.678	0.119	0.070
All pooled	48	1,537	0.075	0.065	0.541	0.114	0.059
***APL1B***							
Bancoumana dry	19	2,005	0.014	0.017	−0.685	0.017	0.015
Bancoumana rainy	12	2,077	0.030	0.032	−0.213	0.046	0.030
Toumani-Oulena	16	2,067	0.042	0.043	−0.079	0.074	0.036
Makouchetoum	12	1,968	0.039	0.047	−0.832	0.070	0.033
All pooled	59	1,902	0.034	0.046	−0.921	0.057	0.031
***APL1C***							
Bancoumana dry	15	2,569	0.006	0.009	−1.308	0.010	0.005
Bancoumana rainy	10	2,569	0.028	0.028	−0.055	0.059	0.021
Toumani-Oulena	16	2,410	0.027	0.027	−0.002	0.050	0.020
Makouchetoum	12	2,393	0.030	0.029	0.231	0.064	0.020
All pooled	53	2,393	0.031	0.025	0.556	0.061	0.024

The Bancoumana dry season collection is almost entirely M form mosquitoes, the Toumani-Oulena and Makouchetoum collections are almost entirely S form mosquitoes, and the Bancoumana rainy collection is a mixture of M and S form. These same parameter estimates are given separately for M form and S form mosquitoes in Table S1 and for *APL1A^1^* and *APL1A^2^* alleles in Table S2.

a Number of alleles sequenced.

b Locus size, in base pairs, excluding insertions and deletions.

c Average number of differences per pair of alleles, per nucleotide.

d Watterson's estimator of the population genetic parameter 4N_e_μ.

e Tajima's *D* test statistic.

f Average number of difference per pair of alleles, per nucleotide, synonymous sites only.

g Average number of difference per pair of alleles, per nucleotide, nonsynonymous sites only.

Cohuet et al. [17] have previously surveyed polymorphism at 109 genes distributed around the *A*. *gambiae* genome, including 72 genes thought to be involved in immune processes. These data can be thought of a genome “null” distribution to which the *APL1* locus can be compared. All three *APL1* genes exhibit greater nonsynonymous diversity than any individual gene in the Cohuet et al. [17] set, which have an average π_non_ of 0.3% and a maximum of 2.1%. When contrasted to the genome-wide polymorphism data set as a whole, the *APL1* genes show a significant excess of amino acid polymorphism in *A. gambiae* and a deficit of nonsynonymous fixations between *A*. *gambiae* and *A*. *arabiensis* (χ^2^_(1)_ = 5.79; *p* = 0.016, where the test is a 2×2 contingency table populated by the counts of synonymous polymorphisms within *A*. *gambiae* and fixations between *A*. *gambiae* and *A*. *arabiensis* in each the set of *APL1* genes and the genome null gene set). The *APL1* genes also show a highly significant excess of polymorphism relative to interspecific divergence at synonymous sites (χ^2^_(1)_ = 7.54; *p* = 0.006). The pattern observed at *APL1* is opposite to the generally observed tendency for mutational differences to accumulate between species and stands in contrast to the slight excess of nonsynonymous fixations between *A*. *gambiae* and *A*. *arabiensis* in genes with immune function, which has been interpreted to reflect adaptive divergence between these species [17]. The excess of diversity and shared polymorphism we observed at both nonsynonymous and synonymous sites in *APL1* is more consistent with adaptive maintenance of variation over evolutionary time [26] or with interspecific hybridization allowing adaptive introgression of *APL1* alleles between species [27].

A more traditional McDonald-Kreitman [28] test contrasting the ratios of synonymous and nonsynonymous polymorphism within *A*. *gambiae* to synonymous and nonsynonymous divergence between *A*. *gambiae* and *A*. *arabiensis* shows no significant departure from homogeneity for either the *APL1* genes or the Cohuet et al. [17] genome null set (*APL1*: P_syn_ = 342, P_non_ = 478, F_syn_ = 4, F_non_ = 11, *G* = 1.45, *p* = 0.23; genome null: P_syn_ = 1967, P_non_ = 731, F_syn_ = 86, F_non_ = 38, *G* = 0.74, *p* = 0.73). The power of these McDonald-Kreitman tests is severely limited, however, by the very small evolutionary divergence between *A*. *gambiae* and *A*. *arabiensis*. The fact that the “outgroup” *A*. *arabiensis* alleles of the *APL1* genes are phylogenetically nested within *A*. *gambiae* alleles instead of falling at the root of the genealogies (Figure S2) violates basic assumptions of the McDonald-Kreitman test [28] and may invalidate it. The results of these tests should therefore be interpreted with extreme caution.

*Anopheles merus* is more distantly diverged from *A*. *gambiae* than is *A*. *arabiensis*, typically exhibiting 4%–11% divergence between the species at the nucleotide level (e.g., [14]). We applied a multilocus HKA test in a maximum-likelihood framework [29] to test the hypothesis that *APL1* genes have a different evolutionary trajectory than a set of 50 immune-related and immune-independent genes for which published data describing polymorphism in *A*. *gambiae* and divergence between *A*. *gambiae* and *A*. *merus* was available [14],[16],[19],[21],[30],[31]. An evolutionary model that hypothesized the three *APL1* genes to be evolving with adaptive maintenance of polymorphism fit the empirical data highly significantly better than the null model that assumed all genes evolve equivalently neutrally (χ^2^_(3)_ = 32.8, *p* = 3.63×10^−7^), with the *APL1* genes estimated to exhibit 12-fold to 35-fold greater diversity than should be expected if they were evolving neutrally. This value may be slightly inflated by the non-independence of polymorphisms introduced by the low level of paralogous gene conversion in the *APL1* genes, but the principal observation of exceptionally high allelic diversity and low interspecific divergence in *APL1* genes is robust and consistent with adaptive maintenance of polymorphism.

### *APL1* Diversity Is Not Due to Degradation or Pseudogenization

The high diversity observed in the *APL1* genes relative to other genes in the genome could in principle arise if *APL1* evolved under low constraint, such that mutations were tolerated as selectively neutral. The weight of the data, however, does not support this hypothesis. If the *APL1* genes were simply accumulating neutral substitutions at a higher rate than most genes, they should be expected to show greater interspecific divergence than other genes in the genome. In fact, the opposite pattern is seen, with *APL1* alleles obtained from species sister to *A*. *gambiae* genealogically nesting within *A*. *gambiae* alleles (Figure S2), consistent with continued segregation of variants that predate the species split. An alternative hypothesis is that the polymorphism in the *APL1* genes is weakly deleterious, permitted to segregate in extant populations due to relatively low selective constraint but prevented by natural selection from drifting to fixation between species. If this were the case, we might also expect to see an overabundance of nonsense mutations abolishing gene function. There are at least 38 insertion-deletion polymorphisms (indels) segregating in the *APL1* genes, assuming a conservative estimate of 11 indels in the repetitive PANGGL region (Figure S1). Only 3 of these 38 indels disrupt reading frame, well below the 13 expected by chance, and each frame-shift is observed in only a single individual in our sample. Similarly, we observed 341 nucleotide polymorphisms segregating in the three *APL1* genes, but only three of these result in premature stop codons (discounting the termination codon that differentiates *APL1A^2^* from *APL1A^1^* alleles, which we assume results in a distinct functional morph of the APL1A protein). One of the premature stops occurs five amino acids before the C-terminus of APL1B, and all three of them are singletons in our sample. On its face, the appearance of even three segregating stop codons may seem surprising, but low-frequency nonsense mutations, presumably existing at mutation-selection balance, are actually observed fairly commonly in population genetic surveys, including those of genes involved in insect defense (e.g., [32]–[34]). Approximately 30% of the codons in *APL1* genes are one mutational step away from becoming a stop codon, and approximately 1/9 of mutations in these codons will yield premature stops. If we assume that loss-of-function *APL1* alleles are recessive and shielded from selection when at low population frequencies, then approximately 3.3% of the polymorphisms observed in the *APL1* genes should be premature stops. This expectation is slightly higher than but broadly consistent with our observed data (1/110 in *APL1A*, 2/114 in *APL1B*, 0/117 in *APL1C*). The fact that all frame-shift and premature stop polymorphisms are observed at estimated allele frequencies of 2% or less indicates that purifying selection operates to retain gene structure and function. Finally, the observation of a recent directional selective event centered on *APL1* in M form mosquitoes (discussed below) indicates that the *APL1* locus is subject to contemporary adaptive evolution.

### Population Substructure at *APL1*

**Figure 2:**
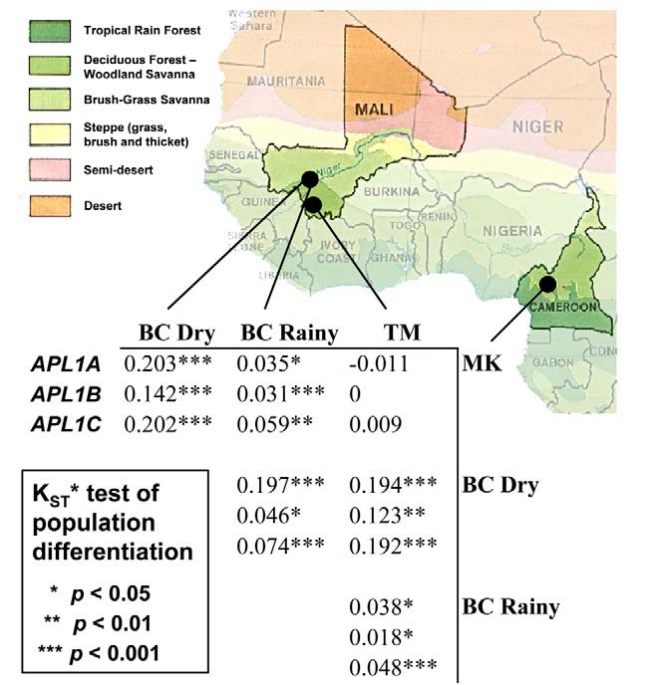
Population differentiation among *A*. *gambiae* collections at the *APL1* locus as estimated by K_ST_*. Statistical significance determined by permutation of alleles among subpopulation pairs [48]. Mosquitoes sampled during the 2005 rainy season from the humid Toumani-Oulena (TM) and Makouchetoum (MK) regions are undifferentiated. These populations are mildly differentiated from a collection drawn from Bancoumana in the 2005 rainy season (BC rainy). All collections are highly differentiated from a collection drawn from Bancoumana in the 2003 dry season (BC dry). The analysis presented in this figure pools all mosquitoes by site and date of collection and does not take into account 2La karyotype or M/S molecular form.

Conspicuously, genetic diversity at *APL1* is not distributed evenly across our population samples, but instead is substructured, perhaps according to microecological factors such as humidity or persistence of standing water. The Toumani-Oulena and Makouchetoum collections, both drawn from humid environments in the 2005 rainy season, are undifferentiated from each other at all three genes (*p*>0.15; Figure 2), but both are mildly differentiated from the 2005 rainy season collection drawn in more arid Bancoumana (*p*<0.05 in all three genes; Figure 2). The Bancoumana collection from the 2003 dry season is highly significantly differentiated from the rainy season collections at all three genes (*p*≤10^−4^ at all three genes for comparisons to Toumani-Oulena and Makouchetoum, *p*≤1.9×10^−2^ when compared to the Bancoumana rainy season collection; Figure 2). Both the 2La chromosomal inversion and the “M” and “S” molecular forms are known to vary geographically and ecologically, so we considered the non-exclusive hypotheses that population differentiation at *APL1* might be attributable to differences in the frequencies of 2La or M/S form.

**Figure 3:**
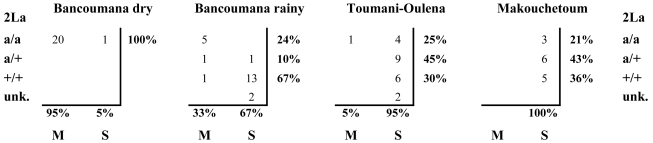
Number of observations of mosquitoes with each 2La inversion karyotype in each molecular form (M and S) over each sample collection. Population frequencies (in percentages) are given in the margins of each table. “Unk.” indicates that 2La karyotype was not determined.

The *APL1* locus lies approximately 1 Mbp inside the distal breakpoint of the polymorphic chromosomal inversion 2La, which has previously been shown to exhibit geographic and microecological variation in frequency. The “inverted” form (2La^a^) of the inversion is more common in drier, more arid environments, and the “standard” orientation (2La^+^) predominates in moister locales [35],[36]. We therefore hypothesized that alternate *APL1* alleles could be associated with the distinct 2La arrangements and that differences in the frequency of the alternate 2La arrangements might underlie the genetic differentiation we observe at *APL1* across our collections. The 2La^a^ orientation is nearly fixed in the Bancoumana collections, but both arrangements are segregating in the Toumani-Oulena and Makouchetoum collections (Figure 3). To test the hypothesis that divergence between 2La^a^ and 2La^+^ chromosomes is responsible for our observed substructure at *APL1*, we measured differentiation in all three *APL1* paralogs after grouping alleles by 2La karyotype irrespective of collection origin. Since it is not possible to identify which of the two homologous chromosomes any *APL1* sequence is derived from in a diploid individual, this analysis can only be conducted using homokaryotypic individuals. The 2La inversion does not segregate in the individuals that were recovered from the Bancoumana dry season collection (all mosquitoes have 2La^a^/2La^a^ homokaryotypes), so we conservatively restricted our analysis of population structure across the inversion to S form mosquitoes from the three rainy season collections. There was mild differentiation between 2La^a^/2La^a^ and 2La^+^/2La^+^ mosquitoes at all three *APL1* paralogs within the S form (*APL1A*: Kst* = 0.059, *p* = 0.016; *APL1B*: Kst* = 0.014, *p* = 0.094; *APL1C*: Kst* = 0.050, *p* = 0.004). Inclusion of all mosquitoes, including the 2La^a^/2La^a^ dry season mosquitoes from Bancoumana in this analysis, results in stronger differentiation at all three *APL1* paralogs, although the inclusion of these mosquitoes conflates the effects of 2La and the “M” and “S” molecular forms (discussed below). No major differences in the amount of *APL1* genetic diversity were observed between 2La^+^/2La^+^ and 2La^a^/2La^a^ homokaryotypes. The differentiation we attribute to 2La is significant and potentially underestimated because our analysis is necessarily restricted to the comparatively small number of homokaryotypic individuals, but it seems to be less severe than the differentiation observed when mosquitoes are categorized by M/S molecular form.

The rDNA polymorphism defining the “M” and “S” molecular forms has also been previously associated with aridity tolerance (reviewed in [25]), and the relative frequency of M and S form mosquitoes is seasonally variable in some *A*. *gambiae* populations [36],[37]. The alternate states of the M/S polymorphism are thought to mark incipiently speciating *A*. *gambiae* subpopulations isolated by pre-mating reproductive barriers (reviewed in [25]). We therefore hypothesized that M/S form might contribute to seasonal genetic substructure at *APL1*. Indeed, 95% of the mosquitoes collected in Bancoumana during the 2003 dry season are M form, while the M form is virtually absent in Toumani-Oulena and Makouchetoum (Figure 3), provisionally supporting the hypothesis that population differentiation at *APL1* might be facilitated by reproductive isolation between the M and S forms. Both M and S form mosquitoes are present at intermediate frequency in Bancoumana during the rainy season (Figure 3), which we hypothesized might explain the intermediacy of this collection in diversity and genetic differentiation from the other subpopulations.

Since both M and S form mosquitoes were sampled during the 2005 rainy season in Bancoumana, we could directly test the hypothesis that the differentiation between M and S molecular forms contributes to subdivision at *APL1*. As expected under this hypothesis, we found that M form mosquitoes from the Bancoumana 2005 rainy season collection are undifferentiated from the Bancoumana 2003 dry season M form mosquitoes but are highly differentiated from the 2005 rainy season S form mosquitoes collected in Toumani-Oulena and Makouchetoum (Table S3). Reciprocally, S form mosquitoes from the 2005 Bancoumana rainy season are undifferentiated from the S form Toumani-Oulena and Makouchetoum collections but are highly significantly differentiated from the 2003 dry season collection, which are M form (Table S3). To further test the hypothesis that isolation between the M and S molecular forms is responsible for the genetic structure we observe at *APL1*, we sequenced *APL1A*, *APL1B*, and *APL1C* in two additional collections of wild *A*. *gambiae*. First, we obtained a second dry season collection of *A*. *gambiae* from Bancoumana, this time collected in 2007. Like the 2003 dry season collection, the 2007 dry season mosquitoes are all M form and are deficient in polymorphism relative to the 2005 rainy season collections. The 2007 dry season mosquitoes are genetically indistinguishable from the 2003 dry season mosquitoes, suggesting these are drawn from the same base population (*p*>0.05 in all genes; Table S3), but as expected, they are highly differentiated from the Toumani-Oulena and Makouchetoum populations (*p*≤0.005 in all genes; Table S3). In a second confirmation, we evaluated a distinct set of M form mosquitoes collected near Bancoumana during the rainy seasons of 1997 and 1999. The *APL1* alleles in these rainy season M form mosquitoes are also genetically indistinguishable from those of the M form 2003 and 2007 dry season mosquitoes (*p*>0.05 in all genes; Table S3) but again are differentiated from the S form Toumani-Oulena and Makouchetoum populations (*p*<10^−3^ in all genes; Table S3). When all mosquitoes from all collections are pooled regardless of population of origin, the S form subpopulation is highly significantly differentiated from the M form subpopulation at all three genes (*p*<10^−4^ at each gene). We therefore conclude that the population substructure we observe in *APL1* genes is primarily due to differentiation between the M form and S form of *A*. *gambiae* and that ecological and season variation contribute only indirectly by influencing M and S prevalence.

### A Recent Selective Sweep in M Form *A*. *gambiae* at *APL1*

**Figure 4:**
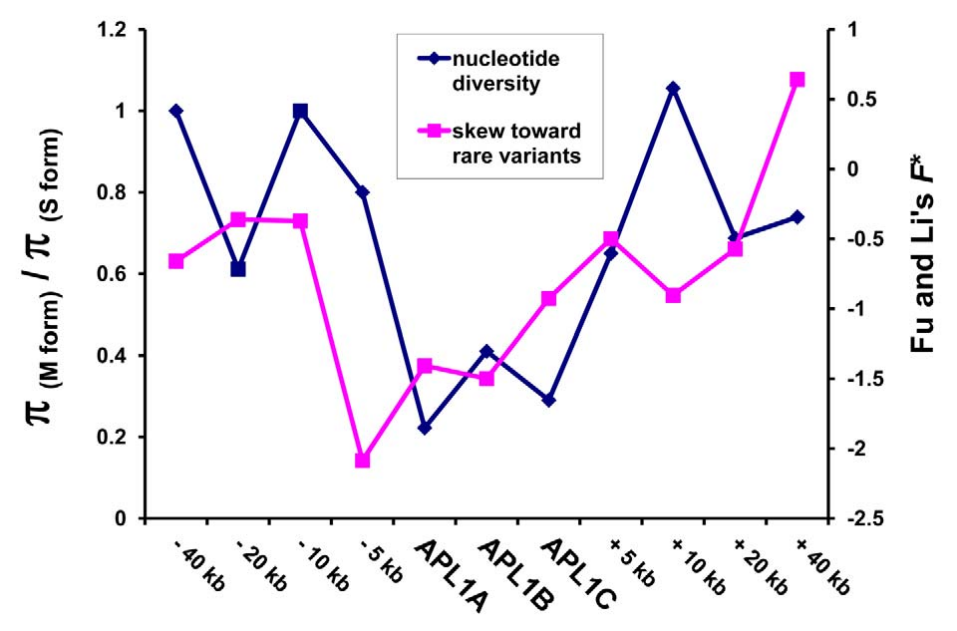
Plot of nucleotide diversity and skew in the site frequency spectrum as a function of physical distance from the *APL1* locus. M form mosquitoes exhibit a sharp drop in polymorphism at the *APL1* locus relative to S form mosquitoes, plotted as the ratio of nucleotide diversity (π) in the M form to diversity in the S form. The M form mosquitoes also exhibit an enhanced skew toward rare variants, indicated by negative values of Fu and Li's F* [41]. The data are consistent with a recent selective sweep at *APL1* in the M form only.

The M form mosquitoes exhibited markedly less genetic diversity at *APL1* than did S form mosquitoes (Table 1, Table S1), raising the possibility that a recent strong selective event may have purged *APL1* genetic variation in the M form population. The classical indications of a recent selective sweep include a deficit of polymorphism [38], and a skew in the site frequency spectrum toward rare genetic variants [39] that can be measured as a negative value of test statistics such as Tajima's *D* (Table 1; [40]) or Fu and Li's F* (Figure 4; [41]) and a deficit of haplotype diversity [42]. The M form population exhibits all three of these characteristics at the *APL1* genes (Table 1, Figure 4, Table 2, Table S1).

**Table 2:** Genetic diversity and haplotype homozygosity in the M and S molecular forms at the *APL1* locus and flanking regions are indicative of selective maintenance of diversity at *APL1* in the S molecular form and a recent selective sweep at *APL1* within the M form.

Position	M Form					EW^f^	S Form					
	bp^a^	π^b^	F*^c^	n^d^	# haps^e^		bp^a^	π^b^	F*^c^	n^d^	# haps^e^	EW^f^
−30 kb	1,181	0.014	−0.660	10	10	0.100	1,181	0.014	−1.667	10	10	0.100
−20 kb	981	0.011	−0.362	10	10	0.100	981	0.018	−0.260	10	10	0.100
−10 kb	1,144	0.014	−0.372	10	10	0.100	1,144	0.014	−0.523	10	10	0.100
−5 kb	1,162	0.008	−2.086	10	10	0.350 (*p* = 0.001)	1,162	0.010	0.584	10	10	0.120
***APL1A***	**1,669**	**0.018**	**−1.407**	**20**	**14**	**0.155 (** ***p*** **<10^−3^)**	**1,537**	**0.081**	**0.568**	**27**	**27**	**0.037**
***APL1B***	**2,005**	**0.016**	**−1.499**	**26**	**17**	**0.172 (** ***p*** ** = 0.002)**	**1,966**	**0.039**	**−1.081**	**34**	**33**	**0.033**
***APL1C***	**2,587**	**0.009**	**−0.927**	**20**	**12**	**0.145 (** ***p*** ** = 0.014)**	**2,393**	**0.031**	**0.404**	**33**	**30**	**0.036**
+5 kb	1,198	0.013	−0.500	10	9	0.120	1,198	0.020	−0.021	9	9	0.111
+10 kb	1,135	0.019	−0.905	10	9	0.120	1,135	0.018	−0.616	10	10	0.100
+20 kb	1,119	0.011	−0.573	10	10	0.100	1,119	0.016	−0.602	10	10	0.100
+40 kb	1,313	0.034	0.642	10	9	0.120	1,313	0.046	−0.178	10	9	0.120

Nucleotide diversity (π) at *APL1* is greatly reduced in the M form relative to the S form with a strong skew toward rare variants (indicated by negative values of F*) that is not observed in the S form (see also Figure 4). Nucleotide diversity at *APL1* is slightly reduced relative to flanking loci within the M form but is greatly elevated relative to flanking loci in the S form. Haplotype diversity is prominently depressed at the *APL1* locus, as indicated by high values of the EW statistic [42] that are significantly incompatible with neutral evolution. No such decrease in haplotype diversity is observed in flanking regions progressively distant from the *APL1* locus in the M form or at any of the S form loci. Most of the haplotypes in M form mosquitoes differ by only one or a few nucleotide substitutions at the *APL1* locus, whereas S form mosquitoes show deeper genealogical structure (see Figure S2).

a Locus size, in base pairs, excluding insertions and deletions.

b Average number of differences per pair of alleles, per nucleotide.

c Skew in the site frequency spectrum, with negative values indicating an excess of rare variants.

d Number of alleles sequenced.

e Number of distinct haplotypes observed.

f Haplotype homozygosity, calculated as the sum of squared observed haplotype frequencies. The use of the EW statistic to contrast the *APL1* genes to the flanking regions is very conservative for the detection of selective sweeps in our experimental framework, as many more alleles were sampled at the *APL1* locus and the physical region surveyed is larger in the *APL1* genes than in flanking regions, both of which allow greater opportunity for recombination to generate distinct haplotypes. *p* values are given only for loci that depart significantly from the neutral expectation.

If *APL1* genes have been the target of a recent selective sweep in M form mosquitoes, we would expect to see the signatures of selection appearing especially prominently at the *APL1* genes themselves and dissipating at progressively distant physical (recombinational) distances away from the locus. To test this, we sequenced loci at approximately 5, 10, 20, and 40 kilobases to either side of the *APL1* locus in both M form and S form Bancoumana mosquitoes. The M form mosquitoes display a prominent dip in diversity relative to diversity in the S form at the *APL1* locus, with variation returning to normal levels by 5–10 kb on either side of *APL1* (Figure 4). The M form mosquitoes also show an enhanced skew toward rare variants and a loss of haplotype diversity at *APL1* that is not observed in S form mosquitoes or in flanking loci (Figure 4, Table 2). Whereas the S form populations show deep genealogical structure at the *APL1* genes and flanking regions, one primary allele has become predominant in each of the *APL1* genes in the M form (Figure S2). This genetic substructure between M and S dissipates with progressive distance to either side of the *APL1* locus, with the M form rapidly regaining deeper genealogical structure and allelic interspersion with the S form (Figure S3). Because these patterns are all restricted to or enhanced at the *APL1* locus, they cannot be due to differences in the demographic history between M and S forms. Collectively, the data provide strong evidence that *APL1* has recently been the target of directional selection specifically in the M form population.

It seems most likely that the inferred selection has operated on variation previously segregating in the M form (as opposed to having acted on a newly occurring mutation) and that the sweep has been only partial. This conclusion is drawn from the fact that the S form is segregating for haplotypes similar to those that predominate in the M form (Figure S2) and that the M form segregates for rare divergent haplotypes that are common in the S form (Figure S2). One explanation for these data could be that continued introgression allows exchange of *APL1* alleles between the M and S forms. Given the degree of divergence among the haplotypes and the low incidence of interbreeding between M and S in the field [25], however, it is more likely that the variation in both forms predates their reproductive isolation and that the partial sweep has purged most of the M form variation at *APL1*. Interestingly, a similar selective event has been reported at the genetically unlinked *Tep1* locus in west African M form mosquitoes [43] (see also [18]). The fact that TEP1 and APL1C physically interact [11]–[12] raises the tantalizing possibility that the two loci have been involved in a coordinate epistatic sweep.

## Discussion

Immune system genes may evolve in complex interplay with pathogens. Elevated diversity in immune-related genes can arise and be maintained over evolutionary time as a consequence of natural selective pressures to combat varied pathogens, but rapid changes in epidemiological pressure can also drive directional selection in defense genes. The *APL1* genes of *A. gambiae* exemplify this complexity of evolution, showing evidence of adaptive maintenance of polymorphism in one subpopulation and strong directional selection in another. In the S form, the *APL1* genes exceed the *A*. *gambiae* genome average diversity by 10-fold and depart markedly from what has been observed in other *A*. *gambiae* defense genes, the majority of which evolve under purifying selection and exhibit little evidence of host-pathogen co-evolutionary dynamics [13]–[17],[19]–[21]. The massively elevated diversity observed in the *APL1* genes is not coupled with an increase in interspecific divergence, suggesting that the dramatic elevation in polymorphism does not arise simply through a high mutation rate or low functional constraint. To the contrary, interspecific divergence is lower at *APL1* than in typical *Anopheles* genes either with or without immune function. The observed pattern of high diversity and low interspecific divergence is more consistent with adaptive maintenance of polymorphism [26]. At the same time, however, we find compelling evidence that a recent selective sweep has acted on the *APL1* locus to favor near-fixation of a single major haplotype in the M form genetic subpopulation, resulting in a sharp local decrease in diversity and a strong skew in the site frequency spectrum toward rare variants. This sweep appears to be coordinate with an independently reported sweep at the *Tep1* gene [43], revealing a rare instance of strong epistatic selection.

While the evolution of *APL1* departs from that of most *Anopheles* defense genes, it bears striking similarity to that of *Tep1*. The APL1C, TEP1, and LRIM1 proteins form a physical complex that activates and stabilizes TEP1 to enact parasite elimination [11],[12], raising the possibility that the complex may evolve coordinately. Like *APL1*, *Tep1* segregates for highly divergent alleles and sustains levels of nonsynonymous diversity approaching that of *APL1* [18], although the level of diversity in *LRIM1* is closer to that typical of *A*. *gambiae* genes [14],[16]. *A. gambiae* alleles from S form mosquitoes are notably paraphyletic with respect to sister species *A*. *arabiensis*, *A*. *quadriannulatus*, and *A*. *merus* at the *APL1*, *Tep1*, and *LRIM1* genes (Figure S2, [16],[18]). While it is not uncommon to find genealogically interspersed alleles of the very closely related (and perhaps occasionally still hybridizing) species *A*. *gambiae* and *A*. *arabiensis* [30], the more distantly related *A*. *merus* typically falls as an outgroup to *A*. *gambiae* genes. As there is little opportunity for ongoing hybridization between *A*. *gambiae* and *A*. *quadriannulatus* or *A*. *merus*, we infer that the incomplete assortment at *APL1*, and perhaps *Tep1* and *LRIM1*, results from continued segregation of alleles that pre-date the formation of these species.

While both the 2La inversion and geographic/ecological sampling location drive mild substructuring of *APL1*, by far the biggest influence on genetic structure at *APL1* is the distinction between M and S molecular forms. The M and S molecular forms are generally reproductively isolated in the field, even when they occur sympatrically [25] as they do at our Bancoumana, Mali, sampling site. Although *APL1* does not lie within any of the previously described “islands” of speciation [44],[45], we find M and S form mosquitoes to be highly significantly differentiated at *APL1*, with strong evidence for a recent partial selective sweep having occurred in the M form. Strikingly, *Tep1* appears to have undergone a similar sweep, also restricted to the M form ([43]; see also [18]). It would be plausible to hypothesize that a coordinate epistatic sweep has impacted the entire APL1C-LRIM1-TEP1 complex in M form mosquitoes. Obbard et al. [14], however, found no evidence for a selective sweep at *LRIM1* in M form mosquitoes collected in Cameroon.

It is unclear why a strong selective event in the *APL1* and *Tep1* genes should be restricted to the M form, although the explanation probably lies in known ecological differences between the forms [25]. Both forms are highly anthropophilic and are competent vectors of human malaria, but they prefer distinct larval habitats, vary in tolerance of aridity, and have only partially overlapping geographic ranges. Although the *APL1*, *Tep1*, and *LRIM1* genes have been characterized as anti-malaria defense factors [4],[7],[8]–[10],[22], it is probable that these are more generic defense molecules. For instance, the observation of Mitri et al. [8] that *APL1C* confers effective defense against rodent malarias *Plasmodium berghei* and *P*. *yoelii* is much more likely to be the result of generic immune activity than of specific co-evolution since *A*. *gambiae* is not naturally exposed to these parasites, and TEP1 has previously been shown to play an important role in phagocytosis of bacteria [46]. Thus, even though *APL1* and interacting genes may be important in defense against malaria parasites, we cannot be certain the evolutionary history of these genes results from selective pressure imposed by *Plasmodium*. Given the ecological differences between M and S form mosquitoes, it is quite likely that distinct pathogens, potentially including pathogens of the larval life stage, could impose distinct selective pressures on the M and S forms, potentially explaining the difference between forms in the evolutionary trajectory of the *APL1* genes.

Our data indicate that functionally variable *APL1* alleles are evolutionarily maintained to combat diverse pathogens, perhaps including but probably not restricted to *Plasmodium* species. Directed, manipulative experiments will be required to test this hypothesis. A more focused selective force seems to have driven a coordinate epistatic sweep at the *APL1* and *Tep1* loci in M form *A*. *gambiae*. While we do not know the proximal agent of selection, the observation underscores the importance of considering M and S form mosquitoes as distinct ecological and genetic entities, even when they are apparently sympatric, with obvious implications for both conventional and genetic control strategies. Our data reveal *APL1* to be one of the few known loci to evolve under both adaptive maintenance of polymorphism and directional selection, and combine with those in [43] to describe a rare instance of epistatic selection on genetically unlinked loci.

## Materials and Methods

### Mosquito Samples

*Anopheles gambiae* were collected inside dwellings from four locations over multiple years. During the 2005 rainy season, samples were taken in July from the agricultural area of Makouchetoum, Cameroon (5°30′N 10°37′W), and in August from the more forested Toumani-Oulena, Mali (10°83′N 7°81′W) and from the village of Bancoumana outside the Malian capital city, Bamako (12°39′N 8°0′W). An additional collection was drawn from N′gabakoro Droit, a village northeast of Bamako, during the dry season in March 2003. For simplicity, this collection is referred to as “Bancoumana-dry” in the article to indicate that it is drawn from the same approximate location but in a distinct time of year as the Bancoumana rainy season collection. In total, we completely sequenced 48 alleles of *APL1A*, 59 alleles of *APL1B*, and 53 alleles of *APL1C* from these initial collections (Table 1), covering more than 6 kb of unique sequence and yielding 451 single nucleotide polymorphisms and 38 insertion/deletion polymorphisms. This sampling should be sufficient to recover the majority of mutations of appreciable frequency in the population [47] (though note that the cited reference assumes a panmictic population, which is certainly not the case with *Anopheles*) and provides sufficient power to detect genetic substructure among populations ([48]; Figure 2, Table S3).

In order to test specific hypotheses regarding population substructure that arose during analysis of the initial data, a second dry season collection was made in Bancoumana itself in 2007 and an additional sample of M form mosquitoes collected in Bancoumana during the rainy seasons of 1997 and 1999 was drawn from pedigrees described in Riehle et al. [4]. The latter pedigree samples are not a completely random sample from the natural population, as they are expected to have undergone some unavoidable selection during their establishment in the lab. We have no reason to suspect, however, that diversity at the *APL1* locus should have been specifically affected during laboratory establishment and maintenance. Six to nine new alleles were sequenced at each gene from these secondary collections, which provided ample power to test our specific hypotheses (Table S3).

*Anopheles quadriannulatus* DNA was obtained from the SKUQUA colony maintained by the Malaria Research and Reference Reagent Resource Center (MR4). *Anopheles arabiensis* were field-collected near Bancoumana in 2003. *Anopheles merus* DNA from mosquitoes of the OPHANSI colony was obtained from MR4.

### DNA Extraction, PCR, and Sequencing

DNA was extracted from the mosquitoes using DNAzol (Invitrogen) or DNeasy kits (Qiagen) under slight modifications to the manufacturers' suggested protocols. PCR primers were designed based on genomic sequence of the *APL1* region of mosquitoes comprising the G3 laboratory colony [7]. Because of the high degree of sequence similarity among the three *APL1* paralogs, primers for this study were designed to flank the coding regions so that each gene could be specifically amplified without cross-amplification of the paralogs. Each paralog was amplified from genomic DNA using iProof high fidelity DNA Polymerase (BioRad). PCR products were run out on a 1% agarose gel, and the amplified products were excised and purified using either the S.N.A.P. gel purification kit or the PureLink get extraction kit (both from Invitrogen). Adenosine tails were added to the purified products by incubation for 20 min at 72°C with PCR buffer, dATP, and Taq polymerase (New England Biolabs). Tailed products were then cloned using the TOPO XL cloning kit (Invitrogen) for sequencing. This strategy of amplifying and cloning entire *APL1* paralogs prior to sequencing allows us to phase polymorphisms within genes, although we do not know the linkage relationships of mutations across paralogs.

Only one of the two alleles at each *APL1* gene was sequenced from any given mosquito in the study. The PCR primers used to screen for colonies containing *APL1B* inserts coincidentally amplified a polymorphic 163 bp deletion in the 3′ UTR, revealing some individual mosquitoes to be heterozygous for that mutation. For these individuals, a coin toss was used to randomly select which allele would be sequenced for inclusion in population genetic analyses. Colonies to be sequenced were grown overnight at 37°C in liquid Luria-Bertani broth supplemented with 20 mg/ml kanamycin, and the plasmids were isolated using the Qiaprep spin miniprep kit (Qiagen). The products were then sequenced directly from the plasmids using the BigDye Terminator Cycle Sequencing Kit v3.1(ABI). The sequences were assembled using Sequencher (Gene Codes Corp.). *APL1* sequences have been deposited into Genbank under accession numbers HQ702785-HQ702849 and HQ860124-HQ860265.

In order the test the hypothesis of a selective sweep at the *APL1* locus in M form mosquitoes, approximately 1 kilobase of sequence data was obtained from 10 M form and 10 S form mosquitoes collected in Bancoumana at noncoding loci approximately 5 kb, 10 kb, 20 kb, and 40 kb to either side of the *APL1* locus, based on the coordinates of “AgamP3” assembly of the reference *A. gambiae* genome sequence. Only 9 S form alleles collected at the position 5 kb 5′ of the *APL1* cluster because the 10th DNA template consistently failed to PCR amplify. None of these loci are located in previously described islands of differentiation between M and S form mosquitoes. Amplification primers were designed to the flanking loci based on the PEST genome sequence [49], and products were sequenced as described above. These flanking sequences have been deposited into Genbank under accession numbers HQ859966-HQ860123.

In order to control for sequencing error, singleton polymorphisms were verified by re-amplification and direct sequencing of heterozygous PCR products or additional independently amplified and cloned products. Genomic DNA was limited for many samples, so whole genome amplification was performed using the GenomiPhi kit (GE Healthcare) prior to singleton validation. Whole genome amplified products were diluted 1∶100, and then 1 ul of diluted amplified DNA was used as template in a 20 ul PCR using primers located outside the gene coding sequence. This full-length amplicon was then used as template in a secondary PCR, in which internally nested primers were used to robustly amplify the gene region containing the singleton to be validated. Unincorporated primers and dNTPs were inactivated from these secondary amplification products by incubation for 60 min at 37°C with ExoI and SAP (both manufactured by USB), with enzymes subsequently inactivated by 10 min incubation at 65°C. Amplification products were then directly sequenced using the BigDye Terminator Cycle Sequencing Kit v3.1 (Applied Biosystems). Across all three *APL1* genes, 470 out of the 581 singleton polymorphisms validated (80.9% validation). This means, prior to correction, our initial cloning and sequencing had an error rate of approximately 3 in 10,000 nucleotides.

PCR amplification of the *APL1A* gene from some individuals occasionally yielded products of unexpectedly small size. DNA sequencing revealed that these bands are similar in sequence to some *APL1A^1^* alleles but carry dramatic genomic deletions that eliminate the presumptive start codon and the entire PANGGL region. If this sequence does indeed code an expressed allele, we infer that translation would initiate with a methionine codon early in the LRR region. We detected some individuals that carried this much shortened *APL1A*-like sequence in addition to more conventional *APL1A^1^* and *APL1A^2^* alleles, suggesting that the shorter *APL1A*-like sequence may be a genomic duplicate. No such *APL1A* duplicate can be found in the completely sequenced *A*. *gambiae* genome [49], and while *APL1A* PCR on some individuals repeatedly yielded the shorter band, other individuals never yielded the shorter product. No individual mosquitoes carried the shortened allele in the absence of any full-length *APL1A* allele. Unfortunately, amplification of this *APL1A*-like duplicate was somewhat unreliable, even across replicate amplifications of the same DNA template, so we are unable to precisely estimate the population frequency of the inferred *APL1A* duplicate. Neither are we able to perform conventional population genetic analyses, due to concerns that our positive amplifications may represent a non-random subset of the naturally existing duplicate alleles. The duplicate alleles that we did sequence are polymorphic for nucleotide variants that are not observed among standard *APL1A* alleles, suggesting that this duplication may be relatively old and evolving independently of *APL1A*.

### Molecular Form and 2La Inversion State

The M/S molecular form of each individual mosquito was determined using the PCR diagnostic developed by Favia et al. [50]. Since *APL1* is located within the 2La chromosomal inversion, 2La karyotype was inferred for each individual using a PCR diagnostic developed by White et al. [51]. M/S and 2La genotyping was performed a minimum of two times on each individual using whole genome amplified DNA template.

### Population Genetic Analyses

Estimates of population diversity based on the number of polymorphic sites (θ_W_) and the average number of pairwise differences among alleles (π) were calculated separately for synonymous and for nonsynonymous sites, as well as for all sites in combination, using DnaSP 5.1 [52]. The normalized difference between these two estimators, Tajima's *D* [40], as well as Fu and Li's F* [41] were also calculated in DnaSP. Haplotype homozyosity (EW) was defined as the sum of squared frequencies of each distinct haplotype observed as described in Zeng et al. [42] and was calculated using a custom script written in C. The distribution of the EW statistic under selective neutrality was determined from 1,000 simulated neutral genealogies of the same sample size and number of segregating sites as each empirical data set. Neutral genealogies were simulated using the program ms [53] conservatively assuming no recombination. The degree of genetic subdivision among pairs of collections was estimated using the K_ST_* statistic [48] as implemented in DnaSP. K_ST_* is a measure of the proportion genetic variation that segregates within a priori subpopulations relative to the total amount of genetic variation across all subpopulations. Significant values of the statistic indicate that individuals from the same subpopulation tend to be genetically more similar to each other than they are to individuals from other subpopulations. The statistical significance of the observed K_ST_* was estimated by comparison to a null distribution of K_ST_* constructed for each pair of populations at each locus by permuting subpopulation identities and re-calculating K_ST_* 10,000 times. Results are reported using the statistic K_ST_* (Figure 2), but the metrics K_ST_ [48] and F_ST_ [54] gave similar results. The maximum likelihood multi-locus HKA test was conducted using mlhka [29] on the 50 gene sets published in [14],[16],[19],[21],[30],[31]. Some of these data sets include multiple *A*. *merus* sequences. In those cases, a single *A*. *merus* sequence was chosen at random for inclusion in the analysis. In instances where the *A*. *merus* sequence was heterozygous, one of the nucleotide states was chosen with 50% probability. Because the true divergence between *A*. *gambiae* and *A*. *merus* is not known, Markov chains were initiated with starting values of 4N_e_ equal to 0.1, 1.0, and 10. Analyses initiated from all three points yielded identical model likelihoods, similar estimates of the selection parameter for the three *APL1* genes, and a maximum likelihood divergence time of 0.35*N_e_ generations. All population genetic statistics were generated excluding polymorphic sites segregating inside insertions and deletions.

## Supporting Information

Figure S1 Alignment of amino acid haplotypes observed in the PANGGL regions of *APL1C* and *APL1A^2^* alleles. Period symbols (.) indicate identity with the residue indicated in the first row. Dashes (-) indicate deleted sequence. The repeated motif TNFGGQ is highlighted in red. The repeated motif PANGGL and related sequences are highlighted in blue. The numbers in the first four columns indicate the number of times each haplotype was observed in the Bancoumana dry, Bancoumana rainy, Toumani-Oulena, and Makouchetoum collections, respectively. The 33 S form mosquitoes carry 19 distinct haplotypes in this protein region, while the 18 M form mosquitoes carry only three haplotypes. The fifth column indicates the molecular form each haplotype was found in. There were no haplotypes found in both molecular forms, and we found no *APL1A^2^* alleles in *A. arabiensis*, *A*. *quadriannulatus*, or *A*. *merus*.
(0.02 MB PDF)

Figure S2 Alleles of the *APL1A*, *APL1B*, and *APL1C* genes show strong genealogical structuring between the M and S molecular forms. A small number of closely related alleles predominate in the M form, whereas the S form shows deeper genealogical structure. The data are consistent with a recent selective sweep that has been restricted to the M form, purging diversity from the M form but not the S form. The plotted genealogies are neighbor joining trees, drawn in MEGA 3.1 [55] using uncorrected p-distance and pairwise-deletion comparisons. Nodes with greater than 50% bootstrap support are indicated. Tips labeled “BC dry” were collected in the 2003 dry season in Bancoumana, Mali; tips labeled “BC rainy” were collected in Bancoumana during the 2005 rainy season; tips labeled “Makouchetoum” were collected during the 2005 rainy season in Makouchetoum, Cameroon; and tips labeled “Toumani-Oulena” were collected during the 2005 rainy season in Toumani-Oulena, Mali.
(0.02 MB PDF)

Figure S3 Alleles 5 kb, 10 kb, 20 kb, and 40 kb to either side of *APL1* gene cluster do not show strong genealogical structuring between the M and S molecular forms. Whereas the *APL1* genes show strong subdivision between M and S and very little diversity within the M form, alleles from M and S form alleles become progressively more genealogically interspersed and the M form shows greater genealogical depth with increasing physical (recombinational) distance from the *APL1* locus. These data indicated that the structuring observed at *APL1* is restricted to that locus and is not a general property of M/S differentiation, consistent with a recent selective sweep at *APL1* in the M form, purging diversity from the M form but not the S form. All mosquitoes in these figures were collected in Bancoumana, Mali. The plotted genealogies are neighbor joining trees, drawn in MEGA 3.1 [55] using uncorrected p-distance. Nodes with greater than 50% bootstrap support are indicated; scale bar indicates nucleotide divergence.
(0.05 MB PDF)

Table S1 Population genetic parameter estimates for M and S form mosquitoes at the three *APL1* paralogs.
(0.05 MB PDF)

Table S2 Population genetic parameter estimates at the *APL1A* locus, considered separately for alleles falling in the *APL1A^1^* and *APL1A^2^* structural classes.
(0.01 MB PDF)

Table S3 Subpopulation differentiation at the *APL1* locus across geographic and temporal samples, structured by M and S molecular form. An insufficient number of M form *APL1A* alleles were sequenced from Bancoumana in the 2005 rainy season to conduct the analysis with confidence. Differentiation is estimated by K_ST_*, with statistical significance (in parentheses) determined through 1,000 permutations of alleles among collections. In all cases, M form mosquitoes are highly significantly differentiated from S form mosquitoes regardless of geographic or temporal origin.
(0.04 MB PDF)

## Abbreviations

LRR: leucine-rich repeat
QTL: quantitative trait locus
