# Supplementary material for: Exceptional Diversity, Maintenance of Polymorphism, and Recent Directional Selection on the APL1 Malaria Resistance Genes of Anopheles gambiae
Source: PLoS Biol. 2011 Mar 8;9(3):e1000600. doi: 10.1371/journal.pbio.1000600 (PMC3050937; doi:10.1371/journal.pbio.1000600)
Supplement: Figure S1 — Alignment of amino acid haplotypes observed in the PANGGL regions of APL1C and APL1A2 alleles. Period symbols (.) indicate identity with the residue indicated in the first row. Dashes (-) indicate deleted sequence. The repeated motif TNFGGQ is highlighted in red. The repeated motif PANGGL and related sequences are highlighted in blue. The numbers in the first four columns indicate the number of times each haplotype was observed in the Bancoumana dry, Bancoumana rainy, Toumani-Oulena, and Makouchetoum collections, respectively. The 33 S form mosquitoes carry 19 distinct haplotypes in this protein region, while the 18 M form mosquitoes carry only three haplotypes. The fifth column indicates the molecular form each haplotype was found in. There were no haplotypes found in both molecular forms, and we found no APL1A2 alleles in A. arabiensis, A. quadriannulatus, or A. merus. (0.02 MB PDF) [file pbio.1000600.s001.pdf]

Supporting Figure S1: Alignment of amino acid haplotypes observed in the PANGGL regions of *APL1C* and *APL1A*^{2} alleles. Period symbols (.) indicate identity with the residue indicated in the first row. Dashes (-) indicate deleted sequence. The repeated motif TNFGGQ is highlighted in red. The repeated motif PANGGL and related sequences are highlighted in blue. The numbers in the first four columns indicate the number of times each haplotype was observed in the Bancoumana dry, Bancoumana rainy, Toumani-Oulena and Makouchetoum collections, respectively. The 33 S form mosquitoes carry 19 distinct haplotypes in this protein region, while the 18 M form mosquitoes carry only 3 haplotypes. The fifth column indicates the molecular form each haplotype was found in. There were no haplotypes found in both molecular forms, and we found no *APL1A*^{2} alleles in *A. arabiensis, A. quadriannulatus*, or *A. merus*.

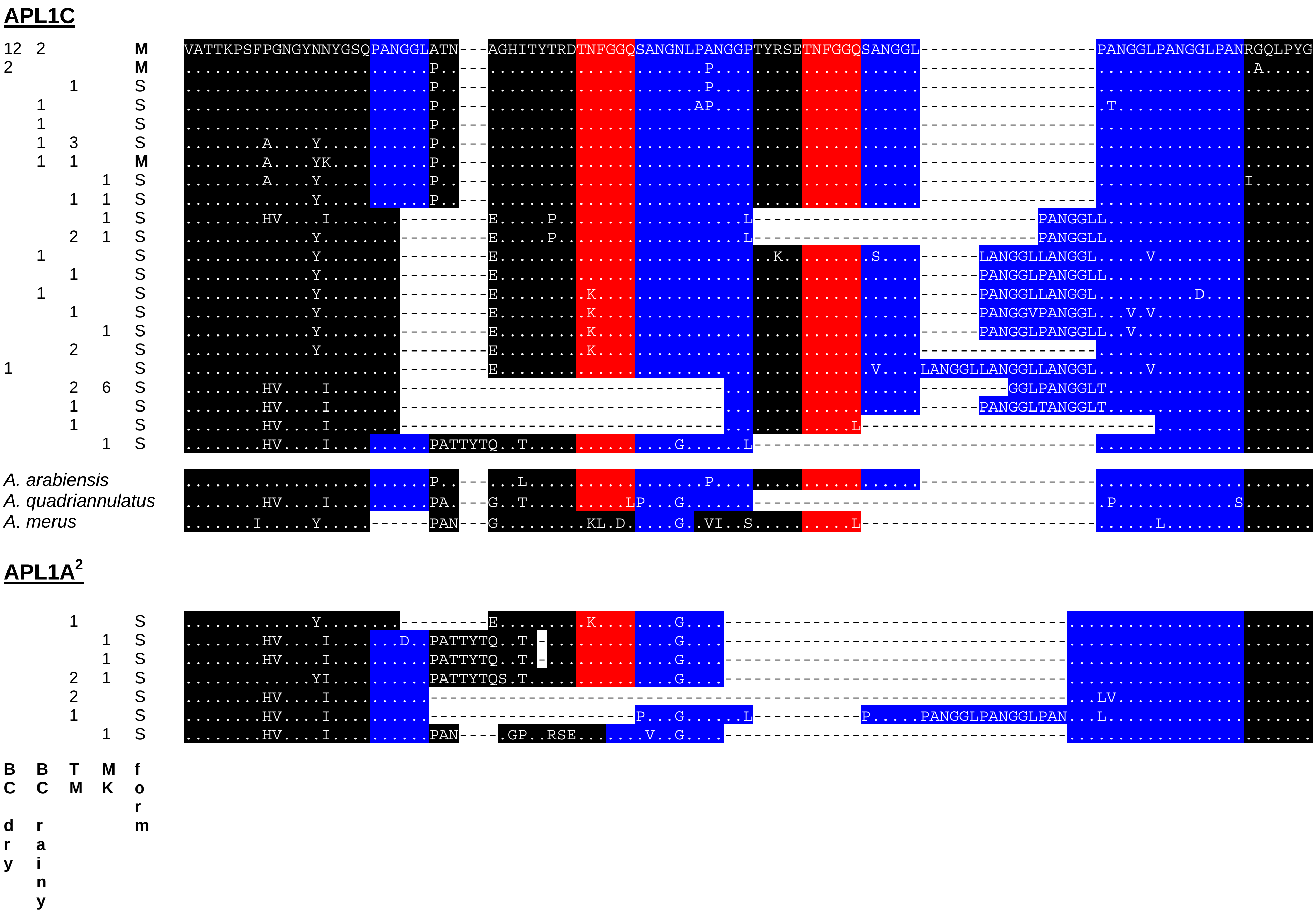
APL1C
VATTKPSFPGNGYNNYGSQPANGGLATN
AGHITYTRDTNFGGQSANGNLPANGGPTYRSETNFGGQSANGGL
PANGGLPANGGLPANRGQLPYG
A. arabiensis
A. quadriannulatus
A. merus
APL1A2
BC dry
BC rainy
TM
MK
form
