# Supplementary material for: Exceptional Diversity, Maintenance of Polymorphism, and Recent Directional Selection on the APL1 Malaria Resistance Genes of Anopheles gambiae
Source: PLoS Biol. 2011 Mar 8;9(3):e1000600. doi: 10.1371/journal.pbio.1000600 (PMC3050937; doi:10.1371/journal.pbio.1000600)
Supplement: Figure S2 — Alleles of the APL1A, APL1B, and APL1C genes show strong genealogical structuring between the M and S molecular forms. A small number of closely related alleles predominate in the M form, whereas the S form shows deeper genealogical structure. The data are consistent with a recent selective sweep that has been restricted to the M form, purging diversity from the M form but not the S form. The plotted genealogies are neighbor joining trees, drawn in MEGA 3.1 [55] using uncorrected p-distance and pairwise-deletion comparisons. Nodes with greater than 50% bootstrap support are indicated. Tips labeled “BC dry” were collected in the 2003 dry season in Bancoumana, Mali; tips labeled “BC rainy” were collected in Bancoumana during the 2005 rainy season; tips labeled “Makouchetoum” were collected during the 2005 rainy season in Makouchetoum, Cameroon; and tips labeled “Toumani-Oulena” were collected during the 2005 rainy season in Toumani-Oulena, Mali. (0.02 MB PDF) [file pbio.1000600.s002.pdf]

Supporting Figure S2: Alleles of the *APL1A*, *APL1B* and *APL1C* genes show strong genealogical structuring between the M and S molecular forms. A small number of closely related alleles predominate in the M form, whereas the S form shows deeper genealogical structure. The data are consistent with a recent selective sweep that has been restricted to the M form, purging diversity from the M form but not the S form. The plotted genealogies are neighbor joining trees, drawn in MEGA 3.1 [54] using uncorrected p-distance and pairwise-deletion comparisons. Nodes with greater than 50% bootstrap support are indicated. Tips labeled “BC dry” were collected in the 2003 dry season in Bancoumana, Mali, tips labeled “BC rainy” were collected in Bancoumana during the 2005 rainy season, tips labeled “Makouchetoum” were collected during the 2005 rainy season in Makouchetoum, Cameroon, and tips labeled “Toumani-Oulena” were collected in during the 2005 rainy season in Toumani-Oulena, Mali.

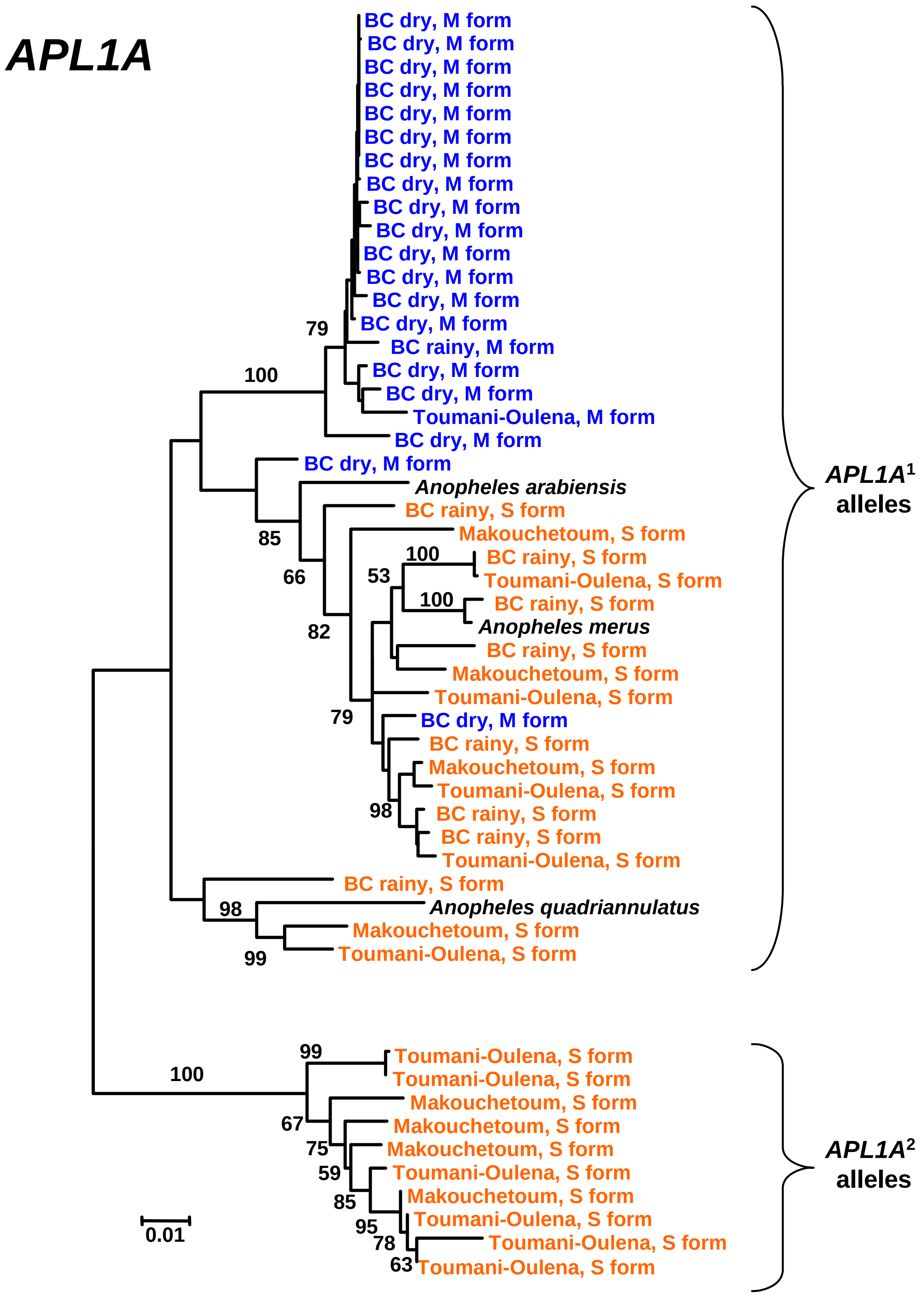

APL1A
BC dry, M form
BC dry, M form
BC dry, M form
BC dry, M form
BC dry, M form
BC dry, M form
BC dry, M form
BC dry, M form
BC dry, M form
BC dry, M form
BC dry, M form
BC dry, M form
BC dry, M form
BC dry, M form
79
BC rainy, M form
100
BC dry, M form
BC dry, M form
Toumani-Oulena, M form
BC dry, M form
BC dry, M form
Anopheles arabiensis
85
BC rainy, S form
Makouchetoum, S form
100
BC rainy, S form
66
Toumani-Oulena, S form
53
100
BC rainy, S form
82
Anopheles merus
BC rainy, S form
Makouchetoum, S form
Toumani-Oulena, S form
79
BC dry, M form
BC rainy, S form
Makouchetoum, S form
Toumani-Oulena, S form
98
BC rainy, S form
BC rainy, S form
Toumani-Oulena, S form
BC rainy, S form
98
Anopheles quadriannulatus
Makouchetoum, S form
99
Toumani-Oulena, S form
APL1A1 alleles
99
Toumani-Oulena, S form
100
Toumani-Oulena, S form
Makouchetoum, S form
67
Makouchetoum, S form
75
Makouchetoum, S form
59
Toumani-Oulena, S form
85
Makouchetoum, S form
95
Toumani-Oulena, S form
78
Toumani-Oulena, S form
63
Toumani-Oulena, S form
0.01
APL1A2 alleles

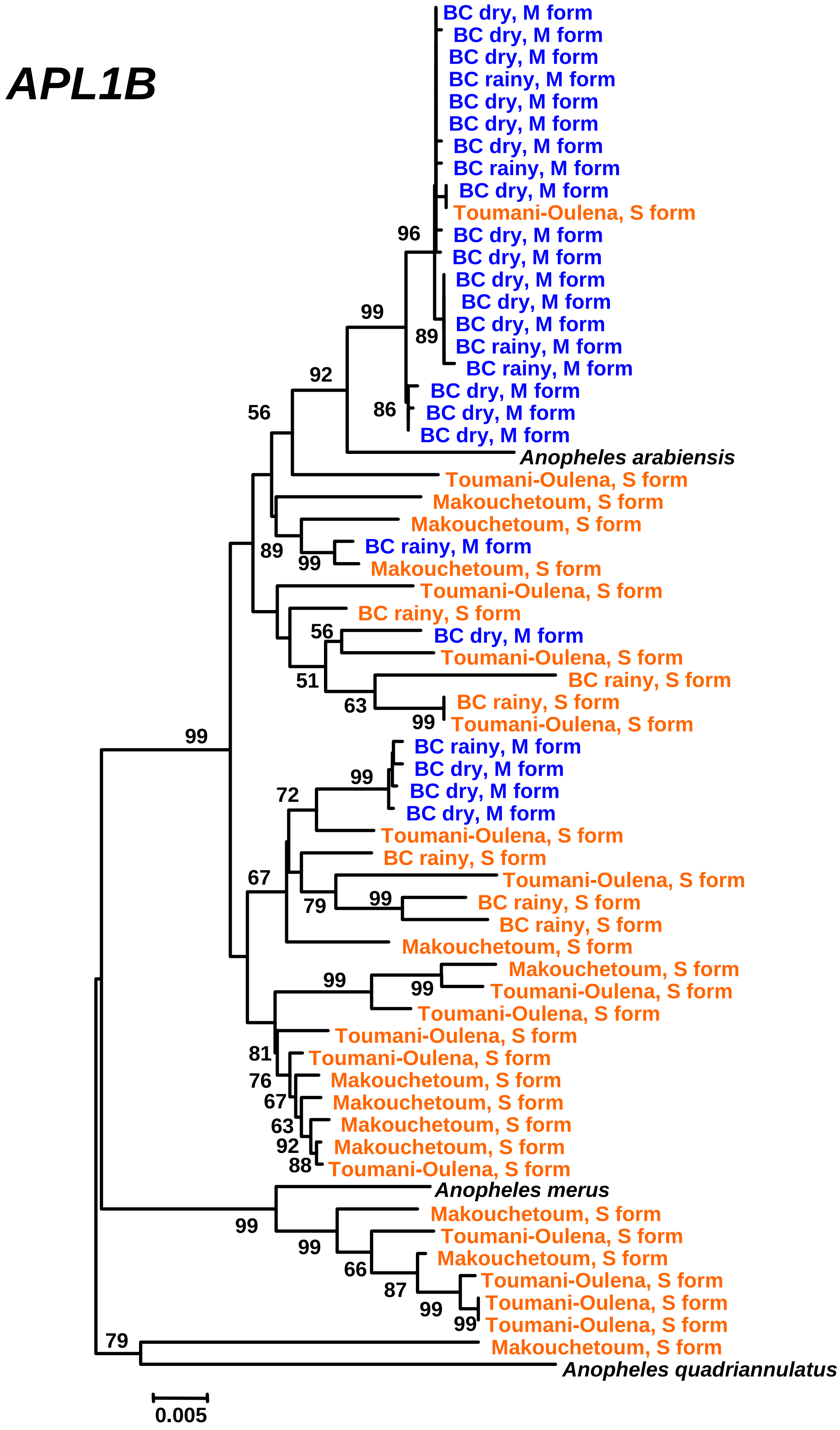
APL1B
BC dry, M form
BC dry, M form
BC dry, M form
BC rainy, M form
BC dry, M form
BC dry, M form
BC dry, M form
BC rainy, M form
BC dry, M form
Toumani-Oulena, S form
BC dry, M form
BC dry, M form
BC dry, M form
BC dry, M form
BC dry, M form
BC rainy, M form
BC rainy, M form
BC dry, M form
BC dry, M form
BC dry, M form
Anopheles arabiensis
Toumani-Oulena, S form
Makouchetoum, S form
Makouchetoum, S form
BC rainy, M form
Makouchetoum, S form
Toumani-Oulena, S form
BC rainy, S form
BC dry, M form
Toumani-Oulena, S form
BC rainy, S form
BC rainy, S form
Toumani-Oulena, S form
BC rainy, M form
BC dry, M form
BC dry, M form
BC dry, M form
Toumani-Oulena, S form
BC rainy, S form
Toumani-Oulena, S form
BC rainy, S form
BC rainy, S form
Makouchetoum, S form
Makouchetoum, S form
Toumani-Oulena, S form
Toumani-Oulena, S form
Toumani-Oulena, S form
Toumani-Oulena, S form
Makouchetoum, S form
Makouchetoum, S form
Makouchetoum, S form
Makouchetoum, S form
Toumani-Oulena, S form
Anopheles merus
Makouchetoum, S form
Toumani-Oulena, S form
Makouchetoum, S form
Toumani-Oulena, S form
Toumani-Oulena, S form
Toumani-Oulena, S form
Makouchetoum, S form
Anopheles quadriannulatus
0.005

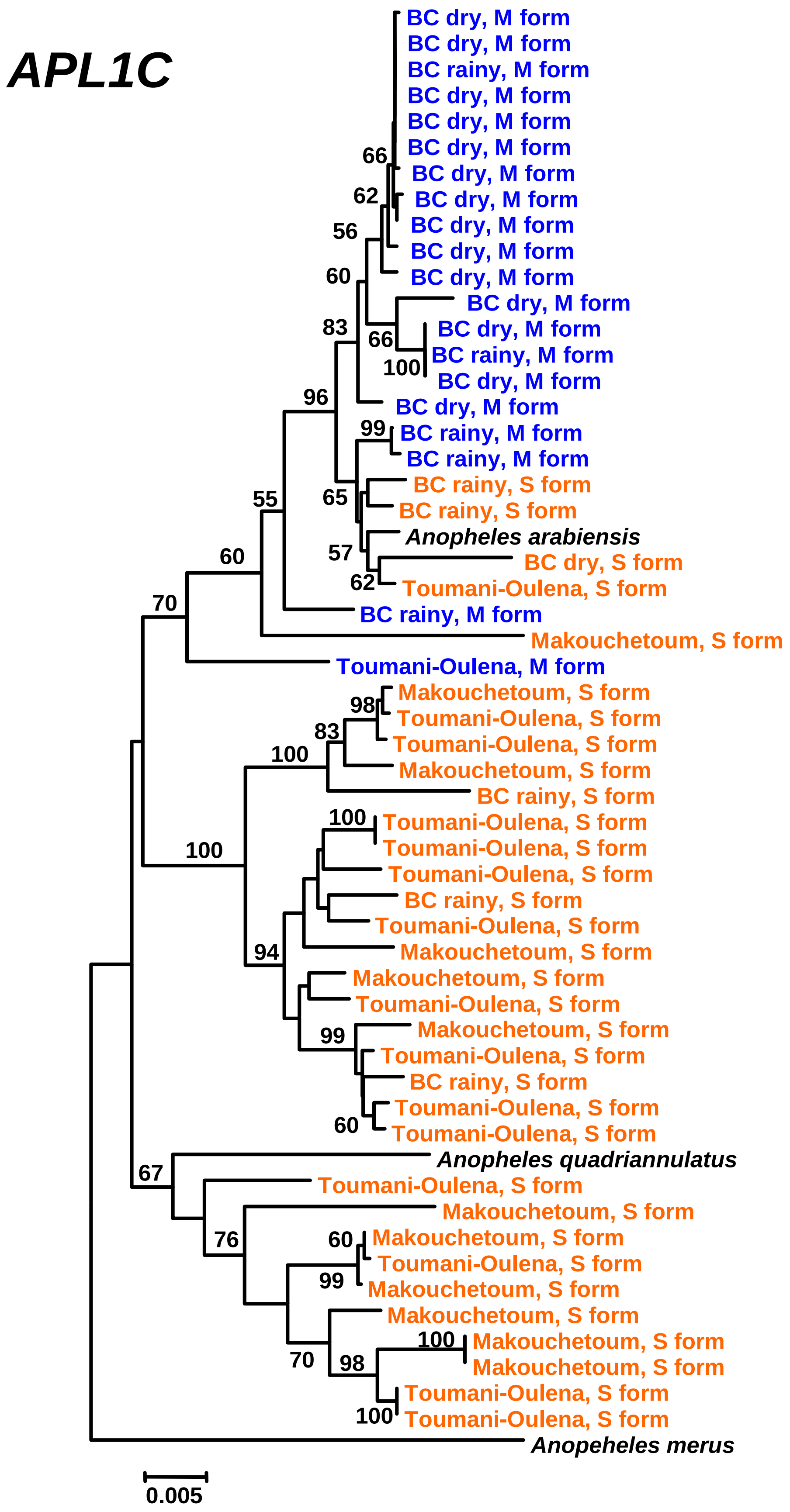
APL1C
BC dry, M form
BC dry, M form
BC rainy, M form
BC dry, M form
BC dry, M form
BC dry, M form
BC dry, M form
BC dry, M form
BC dry, M form
BC dry, M form
BC dry, M form
BC dry, M form
BC dry, M form
BC rainy, M form
BC dry, M form
BC dry, M form
BC rainy, M form
BC rainy, M form
BC rainy, S form
BC rainy, S form
Anopheles arabiensis
BC dry, S form
Toumani-Oulena, S form
BC rainy, M form
Makouchetoum, S form
Toumani-Oulena, M form
Makouchetoum, S form
Toumani-Oulena, S form
Toumani-Oulena, S form
Makouchetoum, S form
BC rainy, S form
Toumani-Oulena, S form
Toumani-Oulena, S form
Toumani-Oulena, S form
BC rainy, S form
Toumani-Oulena, S form
Makouchetoum, S form
Makouchetoum, S form
Toumani-Oulena, S form
Makouchetoum, S form
Toumani-Oulena, S form
BC rainy, S form
Toumani-Oulena, S form
Toumani-Oulena, S form
Anopheles quadriannulatus
Toumani-Oulena, S form
Makouchetoum, S form
Makouchetoum, S form
Toumani-Oulena, S form
Makouchetoum, S form
Makouchetoum, S form
Makouchetoum, S form
Makouchetoum, S form
Toumani-Oulena, S form
Toumani-Oulena, S form
Anopeheles merus
66
62
56
60
83
66
100
96
99
65
55
57
62
60
70
98
83
100
100
100
94
99
60
67
76
60
99
70
98
100
100
0.005
