# Supplementary material for: Exceptional Diversity, Maintenance of Polymorphism, and Recent Directional Selection on the APL1 Malaria Resistance Genes of Anopheles gambiae
Source: PLoS Biol. 2011 Mar 8;9(3):e1000600. doi: 10.1371/journal.pbio.1000600 (PMC3050937; doi:10.1371/journal.pbio.1000600)
Supplement: Figure S3 — Alleles 5 kb, 10 kb, 20 kb, and 40 kb to either side of APL1 gene cluster do not show strong genealogical structuring between the M and S molecular forms. Whereas the APL1 genes show strong subdivision between M and S and very little diversity within the M form, alleles from M and S form alleles become progressively more genealogically interspersed and the M form shows greater genealogical depth with increasing physical (recombinational) distance from the APL1 locus. These data indicated that the structuring observed at APL1 is restricted to that locus and is not a general property of M/S differentiation, consistent with a recent selective sweep at APL1 in the M form, purging diversity from the M form but not the S form. All mosquitoes in these figures were collected in Bancoumana, Mali. The plotted genealogies are neighbor joining trees, drawn in MEGA 3.1 [55] using uncorrected p-distance. Nodes with greater than 50% bootstrap support are indicated; scale bar indicates nucleotide divergence. (0.05 MB PDF) [file pbio.1000600.s003.pdf]

Supporting Figure S3: Alleles 5 kb, 10 kb, 20 kb and 40 kb to either side of *APL1* gene cluster do not show strong genealogical structuring between the M and S molecular forms. Whereas the *APL1* genes show strong subdivision between M and S and very little diversity within the M form, alleles from M and S form alleles become progressively more genealogically interspersed and the M form shows greater genealogical depth with increasing physical (recombinational) distance from the *APL1* locus. These data indicated that the structuring observed at *APL1* is restricted to that locus, and is not a general property of M/S differentiation, consistent with a recent selective sweep at *APL1* in the M form, purging diversity from the M form but not the S form. All mosquitoes in these figures were collected in Bancoumana, Mali. The plotted genealogies are neighbor joining trees, drawn in MEGA 3.1 [54] using uncorrected p-distance. Nodes with greater than 50% bootstrap support are indicated, scale bar indicates nucleotide divergence.

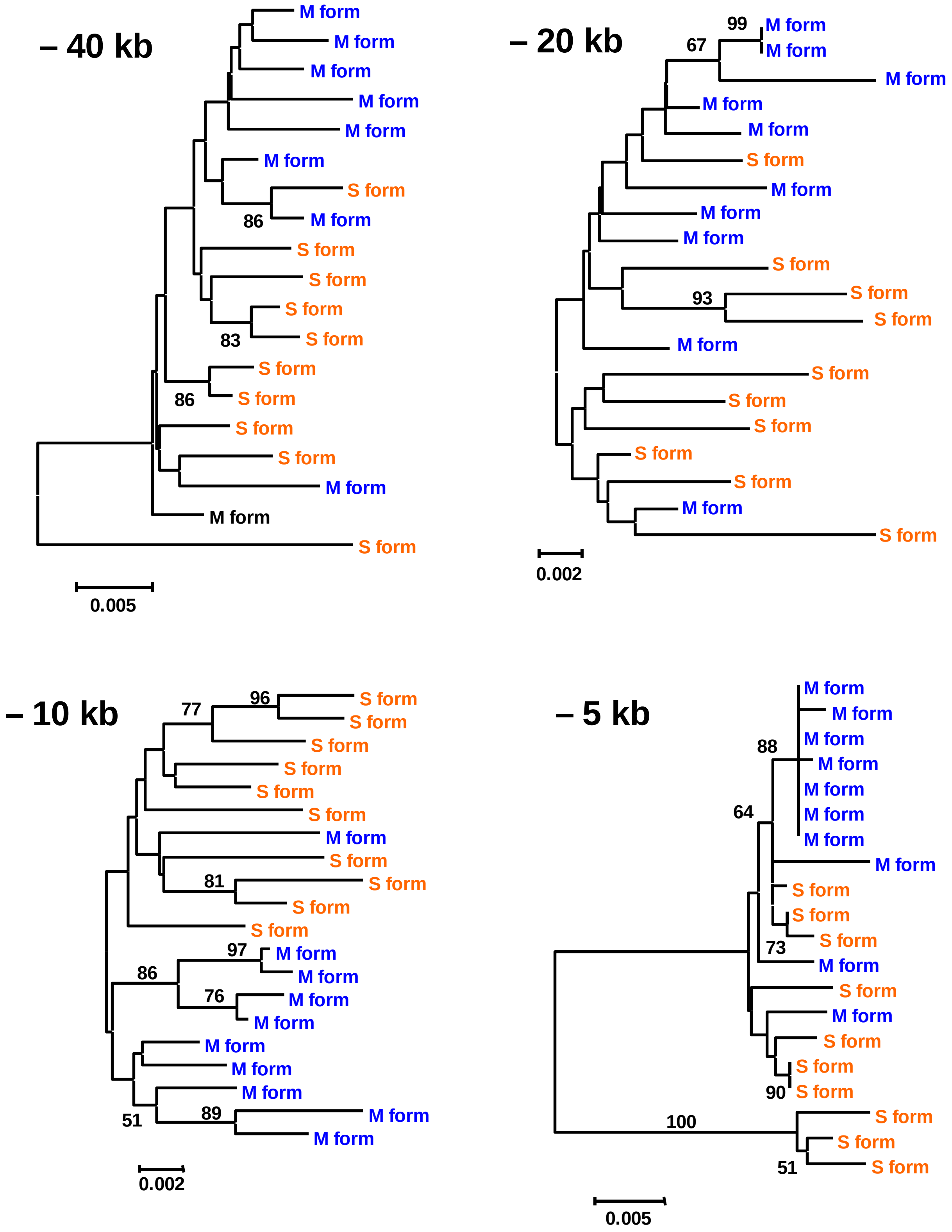

– 40 kb
M form
M form
M form
M form
M form
M form
S form
86
M form
S form
S form
S form
83
S form
S form
86
S form
S form
S form
M form
M form
S form
0.005
– 20 kb
99
M form
67
M form
M form
M form
M form
S form
M form
M form
M form
S form
93
S form
S form
M form
S form
S form
S form
S form
S form
M form
S form
0.002
– 10 kb
77
96
S form
S form
S form
S form
S form
S form
M form
S form
81
S form
S form
S form
97
M form
86
M form
76
M form
M form
M form
M form
M form
51
89
M form
M form
0.002
– 5 kb
M form
M form
88
M form
M form
64
M form
M form
M form
M form
S form
S form
73
S form
M form
S form
M form
S form
S form
90
S form
100
S form
S form
51
S form
0.005

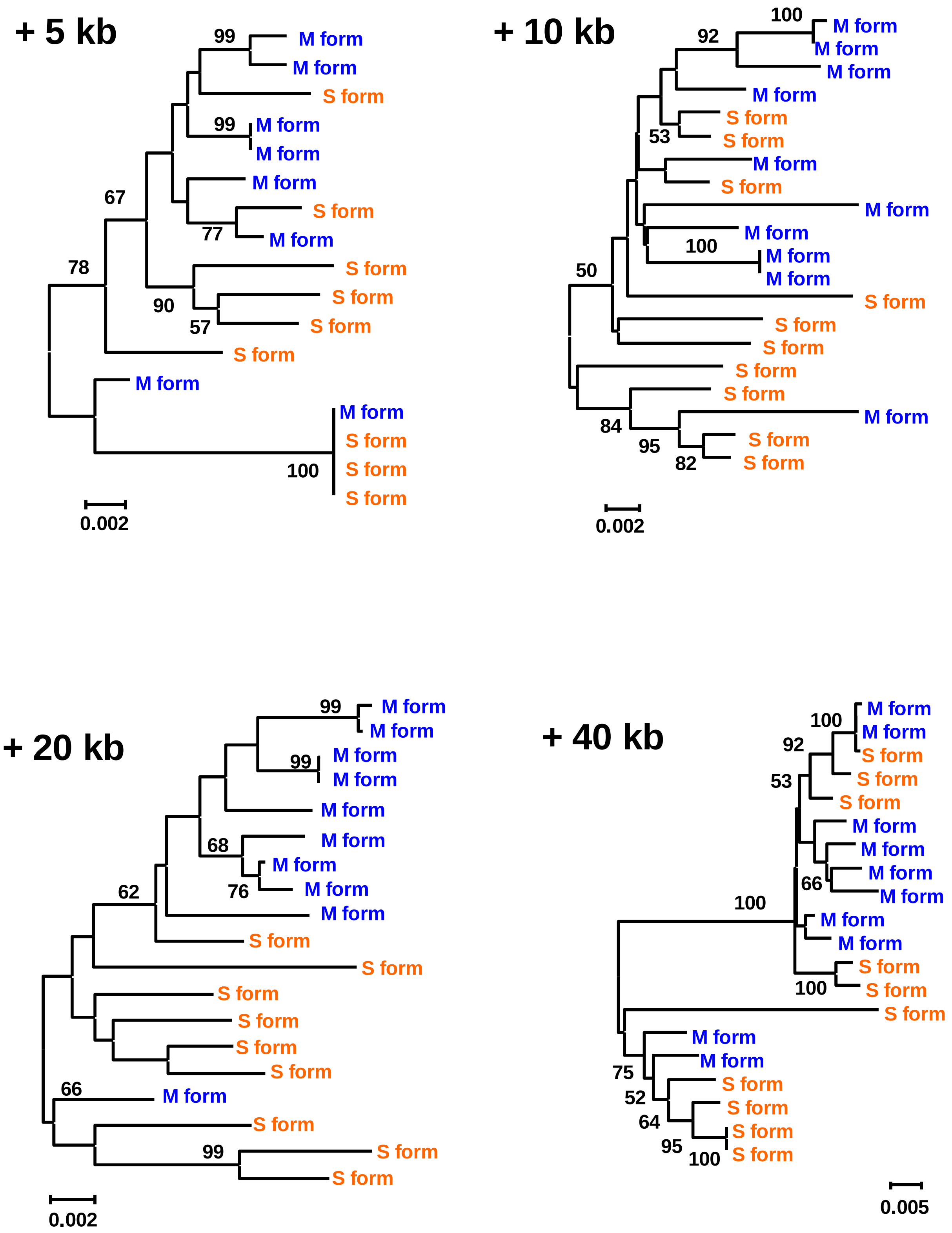

+ 5 kb
99
M form
M form
S form
99
M form
M form
M form
S form
67
77
M form
S form
78
S form
90
S form
57
S form
S form
M form
M form
S form
S form
100
S form
0.002
+ 10 kb
100
M form
92
M form
M form
M form
S form
53
S form
M form
S form
M form
M form
100
M form
50
M form
S form
S form
S form
S form
S form
M form
84
95
S form
82
S form
0.002
+ 20 kb
99
M form
M form
99
M form
M form
M form
68
M form
M form
76
M form
62
M form
S form
S form
S form
S form
S form
S form
66
M form
S form
99
S form
S form
0.002
+ 40 kb
M form
100
M form
92
S form
53
S form
S form
M form
M form
M form
66
M form
100
M form
M form
S form
100
S form
S form
M form
M form
75
S form
52
S form
64
S form
95
100
S form
0.005
