# Supplementary material for: Exceptional Diversity, Maintenance of Polymorphism, and Recent Directional Selection on the APL1 Malaria Resistance Genes of Anopheles gambiae
Source: PLoS Biol. 2011 Mar 8;9(3):e1000600. doi: 10.1371/journal.pbio.1000600 (PMC3050937; doi:10.1371/journal.pbio.1000600)
Supplement: Table S1 — Population genetic parameter estimates for M and S form mosquitoes at the three APL1 paralogs. (0.05 MB PDF) [file pbio.1000600.s004.pdf]

Supporting Table S1: Population genetic parameter estimates for M and S form mosquitoes at the three *APL1* paralogs.

Collection	n[1]	bp[2]	π_{tot}[3]	θ_{tot}[4]	TajD[5]	π_{syn}[6]	π_{non}[7]
APL1A							
M form	20	1669	0.018	0.032	– 1.784	0.034	0.016
S form	28	1537	0.081	0.069	0.703	0.116	0.064
APL1B							
M form	26	2005	0.016	0.021	– 0.816	0.021	0.017
S form	33	1966	0.039	0.048	– 0.691	0.070	0.034
APL1C							
M form	20	2587	0.009	0.012	– 1.037	0.015	0.007
S form	33	2393	0.031	0.028	0.272	0.060	0.022

[1]number of alleles sequenced
[2]locus size, in base pairs, excluding insertions and deletions
[3]average number of differences per pair of alleles, per nucleotide
[4]Watterson's estimator of the population genetic parameter $4N_e\mu$
[5]Tajima's *D* test statistic
[6]average number of difference per pair of alleles, per nucleotide, synonymous sites only
[7]average number of difference per pair of alleles, per nucleotide, nonsynonymous sites only
