# Supplementary material for: Exceptional Diversity, Maintenance of Polymorphism, and Recent Directional Selection on the APL1 Malaria Resistance Genes of Anopheles gambiae
Source: PLoS Biol. 2011 Mar 8;9(3):e1000600. doi: 10.1371/journal.pbio.1000600 (PMC3050937; doi:10.1371/journal.pbio.1000600)
Supplement: Table S2 — Population genetic parameter estimates at the APL1A locus, considered separately for alleles falling in the APL1A1 and APL1A2 structural classes. (0.01 MB PDF) [file pbio.1000600.s005.pdf]

Supporting Table S2: Population genetic parameter estimates at the *APL1A* locus, considered separately for alleles falling in the *APL1A*[1] and *APL1A*[2] structural classes.

Collection	n[1]	bp[2]	π_{tot}[3]	θ_{tot}[4]	TajD[5]	π_{syn}[6]	π_{non}[7]
***APL1A*[1]**							
Bancoumana dry	19	1669	0.019	0.032	– 1.734	0.034	0.016
Bancoumana rainy	9	1665	0.048	0.054	– 0.587	0.086	0.039
Toumani-Oulena	6	1669	0.058	0.062	– 0.434	0.103	0.050
Makouchetoum	4	1669	0.054	0.057	– 0.598	0.076	0.051
all pooled	38	1665	0.048	0.048	0.623	0.104	0.106
***APL1A*[2]**							
Bancoumana dry	0						
Bancoumana rainy	0						
Toumani-Oulena	4	1917	0.017	0.015	0.919	0.150	0.082
Makouchetoum	6	1872	0.023	0.024	– 0.457	0.034	0.018
all pooled	10	1872	0.021	0.023	– 0.542	0.034	0.016

[1]number of alleles sequenced, no *APL1A*[2] alleles were found in the Bancoumana dry population
[2]locus size, in base pairs, excluding insertions and deletions
[3]average number of differences per pair of alleles, per nucleotide
[4]Watterson's estimator of the population genetic parameter $4N_em$
[5]Tajima's *D* test statistic
[6]average number of difference per pair of alleles, per nucleotide, synonymous sites only
[7]average number of difference per pair of alleles, per nucleotide, nonsynonymous sites only
