# Supplementary material for: Exceptional Diversity, Maintenance of Polymorphism, and Recent Directional Selection on the APL1 Malaria Resistance Genes of Anopheles gambiae
Source: PLoS Biol. 2011 Mar 8;9(3):e1000600. doi: 10.1371/journal.pbio.1000600 (PMC3050937; doi:10.1371/journal.pbio.1000600)
Supplement: Table S3 — Subpopulation differentiation at the APL1 locus across geographic and temporal samples, structured by M and S molecular form. An insufficient number of M form APL1A alleles were sequenced from Bancoumana in the 2005 rainy season to conduct the analysis with confidence. Differentiation is estimated by KST*, with statistical significance (in parentheses) determined through 1,000 permutations of alleles among collections. In all cases, M form mosquitoes are highly significantly differentiated from S form mosquitoes regardless of geographic or temporal origin. (0.04 MB PDF) [file pbio.1000600.s006.pdf]

Supporting Table S3: Subpopulation differentiation at the *APL1* locus across geographic and temporal samples, structured by M and S molecular form. An insufficient number of M form *APL1A* alleles were sequenced from Bancoumana in the 2005 rainy season to conduct the analysis with confidence. Differentiation is estimated by K_{ST}*, with statistical significance (in parentheses) determined through 1000 permutations of alleles among collections. In all cases, M form mosquitoes are highly significantly differentiated from S form mosquitoes regardless of geographic or temporal origin.

APL1A	1995 Bancoumana dry (n = 19, M form)	1995 Toumani-Oulena (n = 12, S form)	1995 Makouchetoum (n = 8, S form)
2005 Bancoumana rainy S (n = 8)	0.227 ($p < 10^{-3}$)	0.045 ($p = 0.026$)	0.042 ($p = 0.025$)
1995,1997 Bancoumana rainy M (n = 6)	0.014 ($p = 0.159$)	0.095 ($p < 10^{-3}$)	0.119 ($p < 10^{-3}$)
2007 Bancoumana dry M (n = 6)	0.024 ($p = 0.133$)	0.075 ($p = 0.005$)	0.091 ($p = 0.003$)

APL1B	1995 Bancoumana dry (n = 19, M form)	1995 Toumani-Oulena (n = 15, S form)	1995 Makouchetoum (n = 12, S form)
2005 Bancoumana rainy M (n = 6)	-0.010 ($p = 0.599$)	0.050 ($p = 0.006$)	0.065 ($p = 0.002$)
2005 Bancoumana rainy S (n = 6)	0.241 ($p < 10^{-3}$)	0.012 ($p = 0.110$)	0.026 ($p = 0.005$)
1995,1997 Bancoumana rainy M (n = 7)	-0.019 ($p = 0.856$)	0.103 ($p < 10^{-3}$)	0.134 ($p < 10^{-3}$)
2007 Bancoumana dry M (n = 7)	-0.009 ($p = 0.594$)	0.061 ($p = 0.005$)	0.087 ($p = 0.003$)

APL1C	1995 Bancoumana dry (n = 14, M form)	1995 Toumani-Oulena (n = 15, S form)	1995 Makouchetoum (n = 12, S form)
2005 Bancoumana rainy M (n = 5)	0.065 ($p = 0.046$)	0.082 ($p < 10^{-3}$)	0.085 ($p < 10^{-3}$)
2005 Bancoumana rainy S (n = 5)	0.180 ($p < 10^{-3}$)	0.014 ($p = 0.125$)	0.023 ($p = 0.067$)
1995,1997 Bancoumana rainy M (n = 5)	0.005 ($p = 0.280$)	0.215 ($p < 10^{-3}$)	0.129 ($p < 10^{-3}$)
2007 Bancoumana dry M (n = 7)	0.042 ($p = 0.071$)	0.112 ($p < 10^{-3}$)	0.117 ($p < 10^{-3}$)
